# Supplementary material for: Long-Term Ingestion of Sicilian Black Bee Chestnut Honey and/or D-Limonene Counteracts Brain Damage Induced by High Fat-Diet in Obese Mice
Source: Int J Mol Sci. 2023 Feb 9;24(4):3467. doi: 10.3390/ijms24043467 (PMC9966634; doi:10.3390/ijms24043467)
Supplement: Supplementary file 1 [file ijms-24-03467-s001.zip › ijms-2171755-supplementary.pdf]

Position	RefSeq Number	Symbol	Description
A01	NM_175628	A2m	Alpha-2-macroglobulin
A02	NM_013454	Abca1	ATP-binding cassette, sub-family A (ABC1), member 1
A03	NM_009599	Ache	Acetylcholinesterase
A04	NM_007404	Adam9	A disintegrin and metallopeptidase domain 9 (meltrin gamma)
A05	NM_177034	Apba1	Amyloid beta (A4) precursor protein binding, family A, member 1
A06	NM_018758	Apba3	Amyloid beta (A4) precursor protein-binding, family A, member 3
A07	NM_009685	Apbb1	Amyloid beta (A4) precursor protein-binding, family B, member 1
A08	NM_009686	Apbb2	Amyloid beta (A4) precursor protein-binding, family B, member 2
A09	NM_146104	Aph1a	Anterior pharynx defective 1a homolog (C. elegans)
A10	NM_007467	Aplp1	Amyloid beta (A4) precursor-like protein 1
A11	NM_009691	Aplp2	Amyloid beta (A4) precursor-like protein 2
A12	NM_009692	Apoa1	Apolipoprotein A-I
B01	NM_009696	Apoe	Apolipoprotein E
B02	NM_007471	App	Amyloid beta (A4) precursor protein
B03	NM_011792	Bace1	Beta-site APP cleaving enzyme 1
B04	NM_019517	Bace2	Beta-site APP-cleaving enzyme 2
B05	NM_009738	Bche	Butyrylcholinesterase
B06	NM_007540	Bdnf	Brain derived neurotrophic factor
B07	NM_009810	Casp3	Caspase 3
B08	NM_007609	Casp4	Caspase 4, apoptosis-related cysteine peptidase
B09	NM_007659	Cdk1	Cyclin-dependent kinase 1
B10	NM_007668	Cdk5	Cyclin-dependent kinase 5
B11	NM_183294	Cdkl1	Cyclin-dependent kinase-like 1 (CDC2-related kinase)
B12	NM_009891	Chat	Choline acetyltransferase
C01	NM_013492	Clu	Clusterin
C02	NM_007798	Ctsb	Cathepsin B
C03	NM_009982	Ctsc	Cathepsin C
C04	NM_009983	Ctsd	Cathepsin D
C05	NM_007800	Ctsg	Cathepsin G
C06	NM_009984	Ctsl	Cathepsin L

C07	NM_177821	Ep300	E1A binding protein p300
C08	NM_023913	Ern1	Endoplasmic reticulum (ER) to nucleus signalling 1
C09	NM_008083	Gap43	Growth associated protein 43
C10	NM_010308	Gnao1	Guanine nucleotide binding protein, alpha o
C11	NM_010311	Gnaz	Guanine nucleotide binding protein, alpha z subunit
C12	NM_008142	Gnb1	Guanine nucleotide binding protein (G protein), beta 1
D01	NM_010312	Gnb2	Guanine nucleotide binding protein (G protein), beta 2
D02	NM_013531	Gnb4	Guanine nucleotide binding protein (G protein), beta 4
D03	NM_010313	Gnb5	Guanine nucleotide binding protein (G protein), beta 5
D04	NM_025277	Gng10	Guanine nucleotide binding protein (G protein), gamma 10
D05	NM_025331	Gng11	Guanine nucleotide binding protein (G protein), gamma 11
D06	NM_010316	Gng3	Guanine nucleotide binding protein (G protein), gamma 3
D07	NM_010317	Gng4	Guanine nucleotide binding protein (G protein), gamma 4
D08	NM_010318	Gng5	Guanine nucleotide binding protein (G protein), gamma 5
D09	NM_010319	Gng7	Guanine nucleotide binding protein (G protein), gamma 7
D10	NM_010320	Gng8	Guanine nucleotide binding protein (G protein), gamma 8
D11	NM_010314	Gngt1	Guanine nucleotide binding protein (G protein), gamma transducing activity polypeptide 1
D12	NM_023121	Gngt2	Guanine nucleotide binding protein (G protein), gamma transducing activity polypeptide 2
E01	NM_001031667	Gsk3a	Glycogen synthase kinase 3 alpha
E02	NM_019827	Gsk3b	Glycogen synthase kinase 3 beta
E03	NM_016763	Hsd17b10	Hydroxysteroid (17-beta) dehydrogenase 10
E04	NM_031156	Ide	Insulin degrading enzyme
E05	NM_010514	Igf2	Insulin-like growth factor 2
E06	NM_010554	Il1a	Interleukin 1 alpha
E07	NM_010568	Insr	Insulin receptor
E08	NM_008509	Lpl	Lipoprotein lipase
E09	NM_008512	Lrp1	Low density lipoprotein receptor-related protein 1
E10	NM_008514	Lrp6	Low density lipoprotein receptor-related protein 6
E11	NM_001080926	Lrp8	Low density lipoprotein receptor-related protein 8, apolipoprotein e receptor
E12	NM_010838	Mapt	Microtubule-associated protein tau
F01	NM_010824	Mpo	Myeloperoxidase
F02	NM_001039934	Map2	Microtubule-associated protein 2
F03	NM_144931	Nae1	NEDD8 activating enzyme E1 subunit 1
F04	NM_021607	Ncstn	Nicastrin
F05	NM_026361	Pkp4	Plakophilin 4

F06	NM_008872	Plat	Plasminogen activator, tissue
F07	NM_008873	Plau	Plasminogen activator, urokinase
F08	NM_008877	Plg	Plasminogen
F09	NM_011101	Prkca	Protein kinase C, alpha
F10	NM_008855	Prkcb	Protein kinase C, beta
F11	NM_011102	Prkcg	Protein kinase C, gamma
F12	NM_011103	Prkcd	Protein kinase C, delta
G01	NM_011104	Prkce	Protein kinase C, epsilon
G02	NM_008857	Prkci	Protein kinase C, iota
G03	NM_008859	Prkcq	Protein kinase C, theta
G04	NM_008860	Prkcz	Protein kinase C, zeta
G05	NM_008943	Psen1	Presenilin 1
G06	NM_011183	Psen2	Presenilin 2
G07	NM_008458	Serpina3c	Serine (or cysteine) peptidase inhibitor, clade A, member 3C
G08	NM_009221	Snca	Synuclein, alpha
G09	NM_033610	Sncb	Synuclein, beta
G10	NM_026842	Ubqln1	Ubiquilin 1
G11	NM_025407	Uqcrc1	Ubiquinol-cytochrome c reductase core protein 1
G12	NM_025899	Uqcrc2	Ubiquinol cytochrome c reductase core protein 2
H01	NM_007393	Actb	Actin, beta
H02	NM_009735	B2m	Beta-2 microglobulin
H03	NM_008084	Gapdh	Glyceraldehyde-3-phosphate dehydrogenase
H04	NM_010368	Gusb	Glucuronidase, beta
H05	NM_008302	Hsp90ab1	Heat shock protein 90 alpha (cytosolic), class B member 1
H06	SA_00106	MGDC	Mouse Genomic DNA Contamination
H07	SA_00104	RTC	Reverse Transcription Control
H08	SA_00104	RTC	Reverse Transcription Control
H09	SA_00104	RTC	Reverse Transcription Control
H10	SA_00103	PPC	Positive PCR Control
H11	SA_00103	PPC	Positive PCR Control
H12	SA_00103	PPC	Positive PCR Control
